# Supplementary material for: Multipurpose X-Ray Stage and Its Application for In Situ Poling Studies
Source: Materials (Basel). 2025 Feb 25;18(5):1004. doi: 10.3390/ma18051004 (PMC11901153; doi:10.3390/ma18051004)
Supplement: Supplementary file 1 [file materials-18-01004-s001.zip › materials-3481536-supplementary_final version_REVISED.pdf]

Supplementary Materials

Multipurpose X-Ray Stage and Its Application for In Situ Poling Studies

Antonio Iacomini [1], Davide Sanna [2], Marzia Mureddu [2] , Laura Caggiu [2], Costantino Cau [2,3], Stefano Enzo [2], Edgar Eduardo Villalobos-Portillo [4], Lorena Pardo [5] and Sebastiano Garroni [2,*]

1 Electronic Ceramics Department, Jožef Stefan Institute, 1000 Ljubljana, Slovenia; antonio.iacomini@ijs.si

2 Department of Chemical, Physical, Mathematical and Natural Sciences, University of Sassari, Via Vienna 2, I-07100 Sassari, Italy; dvdsanna@uniss.it (D.S.); m.mureddu6@studenti.uniss.it (M.M.); lcaggiu@uniss.it (L.C.); c.cau1@phd.uniss.it (C.C.); enzo@uniss.it (S.E.)

3 Department of Architecture, Design and Urban Planning, University of Sassari, Piazza Duomo 6, I-07041 Alghero, Italy

4 Alba Synchrotron Light Source, Carrer de la Llum 2-26, 08290 Cerdanyola del Vallès, Spain; evillalobos@cells.es

5 Instituto de Ciencia de Materiales de Madrid, CSIC, C/Sor Juana Ines de la Cruz, 3 Cantoblanco, 28049 Madrid, Spain

* Correspondence: sgarroni@uniss.it

Sintered **Barium Titanate** (BT) pellets were prepared using commercial barium titanate powders (Sigma Aldrich, 99.9% trace metals basis). The powders were finely ground in a mortar with a few drops of a PVA binder solution (3 wt.%) and then were compacted with a hydraulic press. Sintering was conducted at 1300°C for 2h in air.

The sintered density of the as-sintered BT ceramics was determined using the geometric method, measuring the mass with a precision balance, as well as, the height and the diameter of the sintered pellets with a micrometer. The sintered pellet has a final density of 5.58 g/cm^3 which corresponds to about 93% of the theoretical density (6.02 g/cm^3). **Figure S1** shows the XRD patterns of the sintered BT. The samples show the typical tetragonal structure (P4mm s. g.), and no secondary phases are detected. Cell parameters of BT (a = 3.998 Å; b = 4.033 Å) are in good agreement with those found in the literature for pure BT compounds [35].

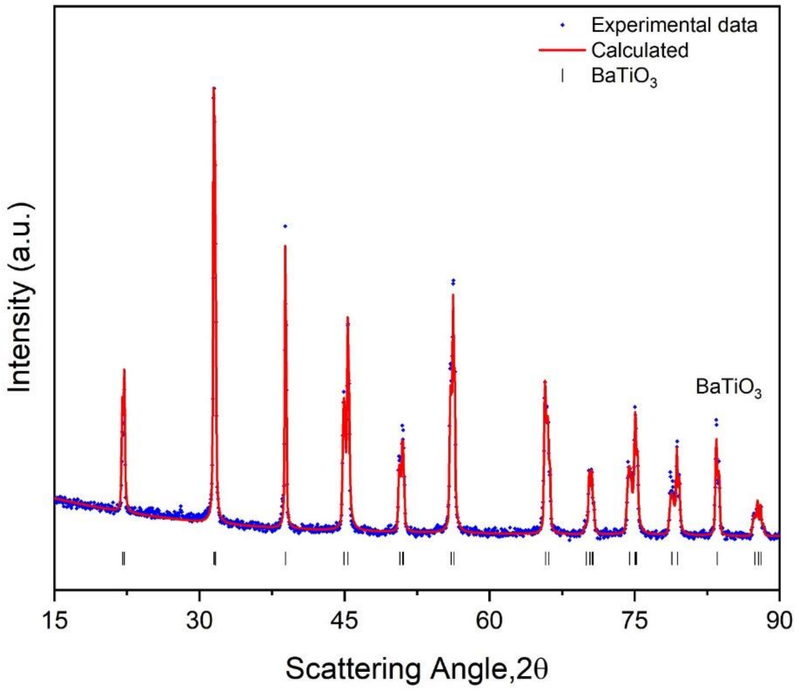

Figure S1. XRD pattern of sintered at 1300°C for 2h. Experimental pattern: blue solid squares. Rietveld refined pattern: red full line.

The SEM micrographs of raw powders and the sintered products are shown in **Figure S2**. Commercial BT powders are characterized by fine and homogeneous particles with dimensions in the order of 1 μm (**Figure S2a**). The sintered product (**Figure S2b**) shows a dense microstructure with predominant transgranular fracture. The porosity detected along the fractured surface justifies the relative density (93%).

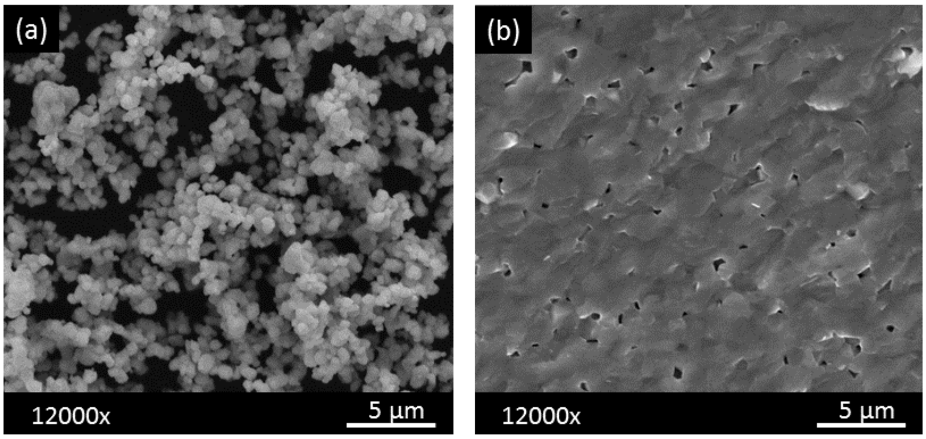

Figure S2. SEM micrograph of (a) commercial BT powders and (b) freshly fractured surface of BT pellet sintered at 1300°C.

$(Ba_{0.92}Ca_{0.08})(Ti_{0.95}Zr_{0.05})O_3$ (BC8TZ5) ceramics were prepared according to the procedure detailed previously using a water-based solid state route [32]. The as-obtained BC8TZ5 pellet presents a bulk density of 5.10 g/cm^3, corresponding to 89% of the theoretical density calculated for the specific composition. The sample showed a pure *P4mm* tetragonal phase without the presence of coexisting polymorphs or secondary phases (see **Figure S3**). The Rietveld refinement of the XRD pattern revealed a tetragonal distortion for the as-produced ceramic (c/a= 1.003 ± 0.001) with c = 4.0202 Å.

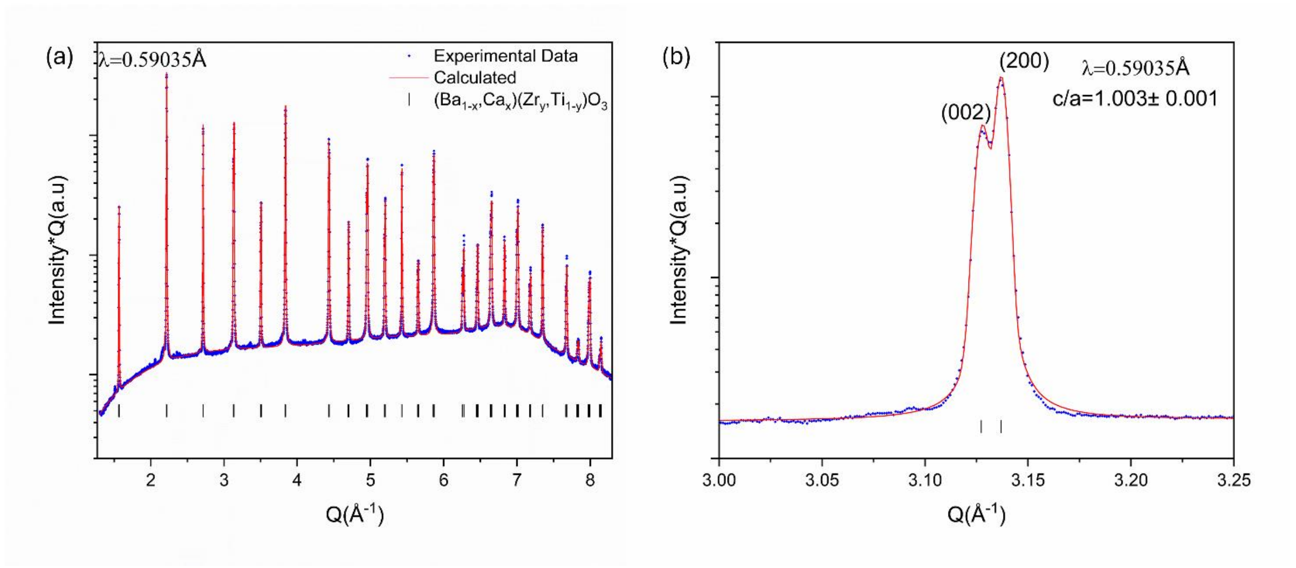

Figure S3. XRD pattern of sintered BC8TZ5 pellet at RT acquired at the NOTOS beamline of ALBA synchrotron. The measurement has been reported in Q(Å^{-1}), reciprocal space. Data points are indicated with blue solid squares. Fitting lines (red full line) of the *P4mm* BC8TZ5 perovskite phase are obtained from Rietveld. (a) Full pattern. (b) magnification of the diagnostic *(002)/(200)* perovskite peak. To facilitate visualization of high-angle peaks, which are much less intense than those at low angles, the pattern has been multiplied by a Q factor.

Figure S4 presents representative SEM images of the fracture surfaces of sintered BC8TZ5 ceramics processed using two-step sintering. The fracture is transgranular, which indicates strong grain boundaries and proper sinterability.

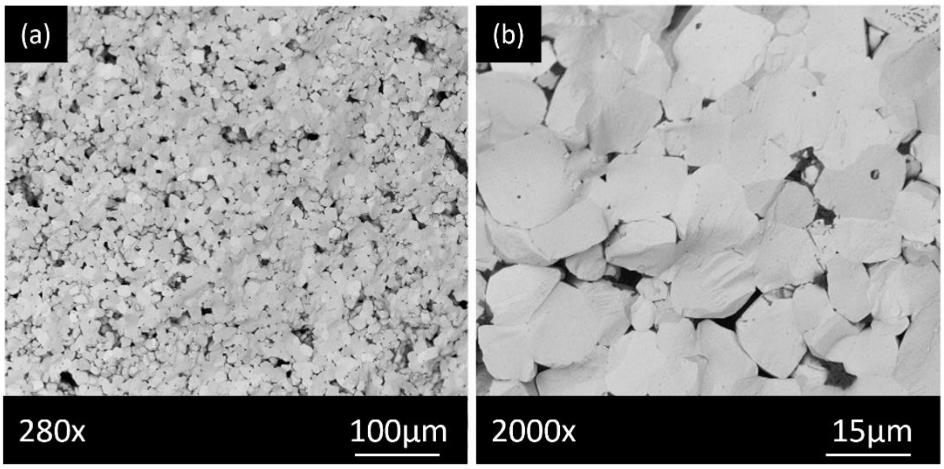

Figure S4. Representative SEM images (backscattering mode) at two different magnifications, of the fracture surfaces of sintered BCZT ceramics obtained according to reference [32].

The crystallographic parameters of the sintered BT samples subjected to thermal treatment from 25°C to 110°C were estimated using the Rietveld refinement method. Detailed results, including lattice parameters, unit cell volume, and phase composition, are summarized in **Table S1.**

**T(°C)**	**s. g**	**a** (Å)	**c** (Å)	**c/a**	**V** (Å^3)
25	*P4mm*	3.996	4.031	1.009	64.367
60	*P4mm*	4.002	4.031	1.007	64.560
80	*P4mm*	4.005	4.029	1.006	64.625
90	*P4mm*	4.007	4.025	1.004	64.626
100	*Pm-3m*	4.012	/	1.000	64.578
110	*Pm-3m*	4.011	/	1.000	64.529

Table S1. Crystallographic information estimated by Rietveld refinement of the sintered BT thermally treated from 25°C up to 110°C.

Figure S5a shows the dielectric permittivity as a function of the temperature for increasing frequency (1, 10, 100 kHz). It is possible to appreciate the clear dielectric anomaly which presents the maximum permittivity at around 106°C, that confirms the ferroelectric-paraelectric phase transition. It is well-known that the dielectric behavior of a normal ferroelectric above the Curie temperature follows the Curie-Weiss law described by the following equation (**Equation S1**) [36, 37]:

$$\frac{1}{\varepsilon} = \frac{(T-T_0)}{C}\ (T > T_c) \qquad \text{(S1)}$$

Where T_0 is the Curie-Weiss temperature, and C is the Curie-Weiss constant. For normal ferroelectric materials, T0 and Tm, which represent the temperature at which the permittivity has its maximum, are very similar. However, ferroelectric ceramics generally present a variable degree of diffusivity which mainly depends on grain size and crystalline defects that cause differences between these two

parameters. In the specific case of the barium titanate under consideration, T_0 is at about 93 °C (**Figure S5b**), which is consistent with the experimental evidence observed by XRD investigation.

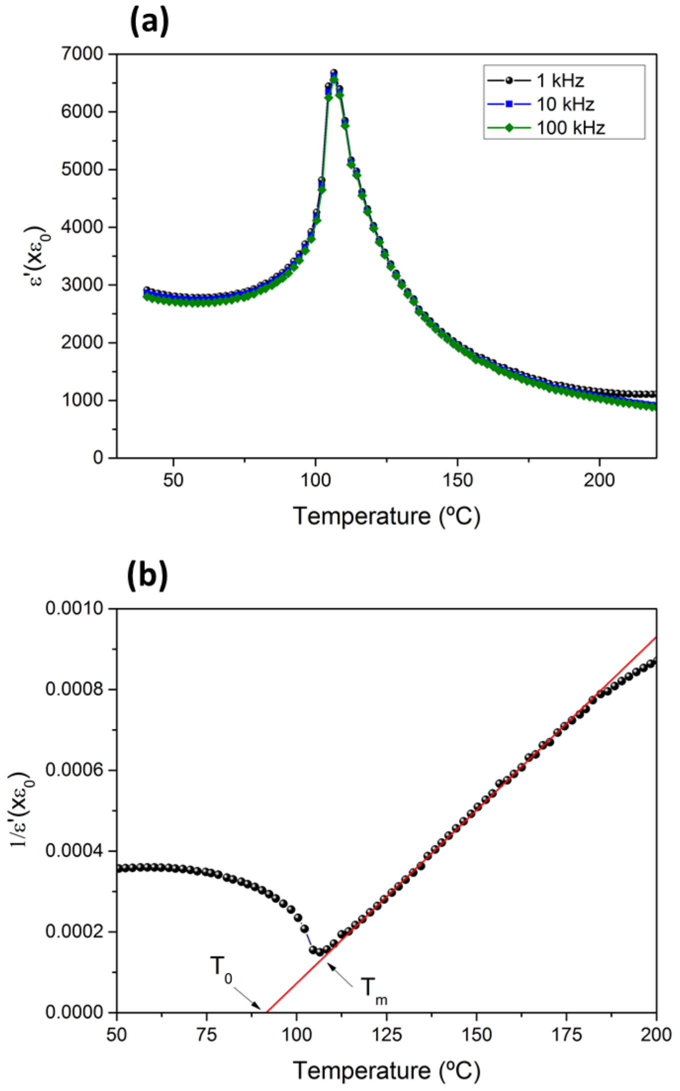

Figure S5. (a) Dielectric permittivity (1, 10, 100 kHz) Vs temperature and (b) inverse dielectric permittivity as a function of temperature at 1 kHz of sintered BT.

In **Table S2**, the I_{200}/I_{002} ratio and the percentage of 90° domains oriented (N) are reported as a function of the poling electric field. These values were obtained from in-situ diffraction experiments carried out at room temperature and 80°C, providing insights into the domain orientation behavior under different conditions.

**Electric field (kV/cm)**	**I_{200}/I_{002} (R.T)**	**N % (R.T)**
0 (ref. state)	1.60(R)	0
4	1.53(r_1)	1
8	1.20(r_2)	7
12	0.94(r_3)	13
16	0.86(r_4)	15
Poled state (no field)	1.23(r_4^*)	6

Table S2. I_{200}/I_{002} ratio and percentage of 90° domains oriented (N) as a function of the poling electric field. The *in-situ* diffraction experiments were conducted at room temperature and 80°C.

Supplementary references

[35] R.H. Buttner, E.N. Maslen, Structural parameters and electron difference density in BaTiO3, Acta Crystallogr. Sect. B 48 (1992) 764–769. https://doi.org/10.1107/S010876819200510X.

[32] M. Mureddu, J.F. Bartolomé, S. Lopez-Esteban, M. Dore, S. Enzo, Á. García, S. Garroni, L. Pardo, BaZrO3-BaTiO3-CaTiO3 piezoceramics by a water-based mixed-oxide route: Synergetic action of attrition milling and lyophilization, J. Eur. Ceram. Soc. (2023).

https://doi.org/10.1016/j.jeurceramsoc.2023.12.037.

[36] X. Zhang, C. Lei, K. Chen, Ferroelectric 90° domain evaluation in tetragonal Pb(Mg 1/3Nb 2/3)O 3-PbTiO 3 ceramics, J. Am. Ceram. Soc. 88 (2005) 335–338. https://doi.org/10.1111/j.1551-2916.2005.00101.x.

[37] A. Iacomini, S. Garroni, G. Mulas, S. Enzo, L. Cappai, M. Mureddu, C. Cau, Á. García, L. Pardo, Processing, phase evolution and electrical properties of “lead free” KNN–BF–CuO eco-piezoceramic from mechanochemically activated precursors, Open Ceram. 9 (2022). https://doi.org/10.1016/j.oceram.2022.100247.
